# CircMYC promotes proliferation, migration, invasion and inhibits apoptosis of small cell lung cancer by targeting miR-145/ Matrix Metallopeptidase 2 axis

**DOI:** 10.1080/21655979.2022.2062978

**Published:** 2022-04-20

**Authors:** Xi Yang, Lianqin Tao, Yani Xu, Sujian Li, Weiwei Yang, Lijuan Wang, Junfei Zhu

**Affiliations:** Department of Respiratory and Critical Care Medicine, Taizhou Central Hospital (Taizhou University Hospital), Zhejiang Province, China

**Keywords:** SCLC, circMYC, miR-145, MMP2, cell proliferation

## Abstract

Circular RNAs (circRNAs) are involved in the carcinogenesis of lung cancer. Human MYC gene is highly expressed in melanoma, multiple myeloma, and nasopharyngeal carcinoma. We aimed to investigate the role of circMYC in small cell lung cancer (SCLC). The expression of cirMYC in SCLC tissues and cells were examined. Functional studies were performed to evaluate the roles of circMYC in SCLC cells. Luciferase reporter gene assay, RNA pull-down assay, and rescue experiments were performed to evaluate the regulatory relationship between circMYC and miR-145, and MiR-145 and MMP2 mRNA. We found that CirMYC was highly expressed in SCLC tissues and cells. Knockdown of circMYC could inhibit cell proliferation, migration, invasion, and induce apoptosis. CircMYC targeted miR-145 and miR-145 targeted MMP2 (Matrix Metallopeptidase 2) mRNA. Our data indicated that circMYC upregulates the expression of MMP-2 by inhibiting miR-145, which functions to promote the proliferation, migration, and invasion and inhibit the apoptosis of SCLC. These findings suggest that targeting circMYC/miR-145/MMP-2 could serve as a potential therapeutic strategy for SCLC treatment.

## Graphical abstract

**Figure:**
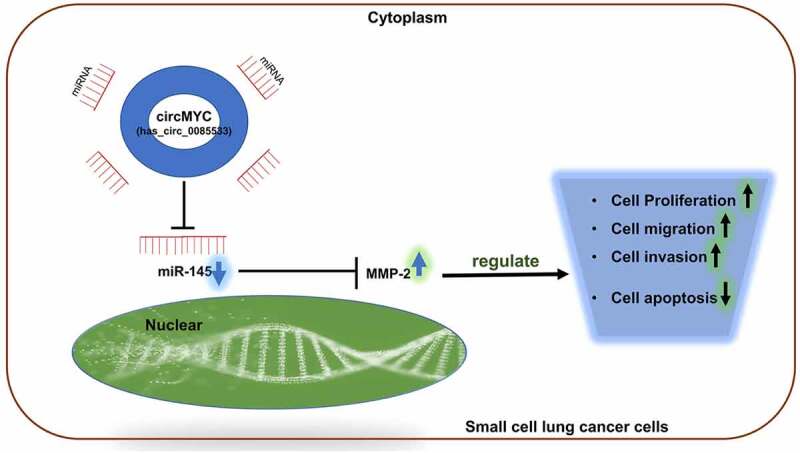

## Highlights

- circMYC is highly expressed in SCLC tumour and cells.
- High circMYC expression is associated with a poor prognosis in SCLC patients.
- CircMYC is essential for the malignant phenotype of SCLC cells.
- CircMYC maintains the high expression level of MMP2 by targeting miR-145.

## Introduction

Lung cancer remains as the leading cause of cancer-related death worldwide and can be divided into two types: small cell lung cancer (SCLC) and non-small cell lung cancer (NSCLC) [1]. Despite many recent advances in the screening, early diagnosis and treatment of lung cancer, the prognosis of this disease is still not optimistic with a very poor survival rate [2,3]. Lung cancer has become a longstanding challenge to global public health. Therefore, it is necessary to deeply investigate the molecular mechanism of lung cancer and identify the potential biomarkers and therapeutic targets to facilitate early and accurate diagnosis and timely treatment for this disease [4].

Non-coding RNAs (ncRNAs) are RNAs without coding potential, which include micro RNAs (miRNAs), long non-coding RNAs (lncRNAs), and circular RNAs (circRNAs). CircRNAs are a unique class of non-coding RNAs with a closed circular structure [5,6]. A growing body of evidence suggests that circRNAs play important roles in many biological processes including cell proliferation, invasion, and cell differentiation [7–10]. In 2010, Poliseno et al. first proposed the hypothesis of the interaction between protein-coding messenger RNAs (mRNAs) and ncRNAs [7,8] and circRNAs could act as ceRNAs [11,12]. Owing to the advantages of abundance, conservation, stability, and high specificity in serum, plasma, and other body fluids [13,14], circRNAs hold great potential as noninvasive blood-based biomarkers in cancer management [15,16]. A recent meta-analysis of the diagnostic efficacy of circRNAs in lung cancer tissues and blood suggested that circRNAs have diagnostic potential in the Chinese lung cancer population [17].

Human circMYC (hsa_circ_0085533) is derived from the MYC gene. The MYC family oncoproteins are believed to regulate the expression of genes governing cellular growth, differentiation, and programmed cell death, and overexpression of MYC is observed in the vast majority of human malignancies [18]. CircMYC expression levels have been suggested to be of prognostic importance in several types of cancer, and the overexpression of circMYC promotes cell proliferation and reduces radiosensitivity in tumor cells [19]. Previous studies have shown that circMYC is highly expressed in melanoma, multiple myeloma, and nasopharyngeal carcinoma, and plays an oncogenic role [20–22]. However, the mechanism underlying the functional roles of circMYC in SCLC remains unclear.

In the current study, we hypothesized that circMYC may function as an important regulator for the malignant phenotype of SCLC. We investigated the expression pattern of circMYC in SCLC tumor tissues and para-cancerous tissues, as well as SCLC cell lines. We further performed circMYC knockdown and functional assays to validate the role of circMYC in the carcinogenesis of SCLC and investigated its regulatory mechanism. These findings suggest that targeting circMYC/miR-145/MMP-2 could serve as a potential therapeutic strategy for SCLC treatment.

## Materials and methods

### Collection of tissue samples

Fifty SCLC tissues and matched adjacent normal lung tissue samples were collected from patients with SCLC undergoing surgical resection with complete data from Taizhou Central Hospital Affiliated to Taizhou College. Surgical specimens were immediately stored in liquid nitrogen until use. All patients had a pathohistological diagnosis of SCLC and provided the written informed consent. This study was approved by the ethics committee of Taizhou Central Hospital Affiliated to Taizhou College。

Inclusion criteria: Subjects who were able to provide written informed consent to participate in the study; Subjects had no other concurrent malignant tumors; Subjects with primary tumors diagnosed as SCLC. Exclusion criteria: Subjects who were undergoing chemotherapy; subjects with blood-related diseases and infectious diseases; subjects who had other chronic diseases; subjects with benign tumors.

## Cell culture and transfection

Four human SCLC cell lines (DMS-53, H446, SHP-77 and H69 obtained from the National Collection of Authenticated Cell Cultures, China) and the human bronchial epithelial cell line 16-HBE (ATCC, USA) were used in this study. siRNAs for circMYC and miR-145 inhibitor were synthesized by RiboBio Company (Guangzhou, China). Transfection of oligonucleotide and plasmid were performed using the Lipofectamine™ 3000 transfection reagent (Invitrogen, USA) according to the manufacturer’s protocols.

## Vector construction and site directed mutagenesis

Luciferase reporter vectors were constructed based on the pGL3 vector as follows: circMYC wild-type luciferase reporter vector WT-circMYC, MMP2 mRNA wild-type luciferase reporter vector WT-MMP2. Takara site directed mutagenesis kit was used to construct the corresponding mutant luciferase reporter vectors: MT-circMYC and MT-MMP2. MMP2 overexpression vector was constructed on the basis of the pcDNA3.1 vector.

## Nucleocytoplasmic fractionation

DMS-53 and SHP-77 cells were plated into 6-well plate at 2 × 10^5^ cells/well, and cells were digested with 0.25% Trypsin when they were at 80% confluence, centrifuged at 2,000 × g for 2 min to recover cytosolic and nuclear RNA, respectively, according to the nucleocytoplasmic Isolation Kit procedure, and the concentration and quality of RNA were determined by nanodrop 2000. The RNA was divided into RNase digested and non-digested fractions. One microgram RNA was digested with 1 U of RNase for 10 min at 37°C. Subsequently, RNA was purified by phenol/chloroform and ethanol precipitation and reverse transcribed to cDNA, and the experiments were repeated three times.

## qRT-PCR

Total RNA was isolated by TRIZOL Reagent (Invitrogen, USA) following the manufacturer’s protocol. RNA samples were reversely transcribed by cDNA Reverse Transcription Kit (Applied Biosystems, USA). The Fast start Universal SYBR Green Master (Roche, USA) was applied for the quantitative RT-PCR. The relative fold changes of RNAs were analyzed by using the 2^−ΔΔCT^ method.

Quantification of miR-145: RNA extraction, reverse transcription, and PCR reactions were performed with the protocol for TaqMan miRNA assays provided by ABI, using rnu6b as internal reference for relative quantification, with three replicate wells set for each group. All primers are listed in Table 1.

**Table 1.** Sequences of qRT-PCR primers

	5’-3’
circMYC	5’-ACTGCGTTTAAACAGAAATCACCT-3’ 5’-GACCAGCAAAATCTGTCTTCGT-3’
miRNA-145	5’-GTCCAGTTTTCCCAGGAATCCCT-3’
	R: 5’-GTCCAGTGCCCATTGGCCTAATCT-3’
PCNA	5’-TTACAGTGACCAACACCTCTAATGCCCCA-3’
Snail1	5’-TTCCAACACCTACAGTGACTAATGCCCCA-3’ 5′-GCGCCCGTCGTCCTTCTCGTC −3′
	5′-CTTCCGCGACTGGGGGTCCT-3′
MMP2	5′-ACTGTTGGTGGGAACTCAGAAG-3′ 5′-CAAGGTCAAT GTCAGGAGAGG-3′
GAPDH	5′-TGTGTCCGTCGTGGATCTGA −3′ 5′-TTGCTGTTGAAGTCGCAGGAG −3′

## Luciferase reporter experiments

Sequences containing the wild-type binding site and the sequence with mutated binding site were cloned into the pGL3-vector expressing firefly luciferase (Promega, E1330). The reporter plasmid and Renilla luciferase (hRlucneo) control plasmid were co-transfected into cells with either miRNA-145 mimic or miR-NC using Lipofectamine 3000 reagent according to the manufacturer’s instructions. Forty-eight hours after transfection, the relative luciferase activities were measured using the Dual-Luciferase Reporter Assay Kit (Promega, E1910) on a luminescence microplate reader. The relative firefly luciferase activity in the reporter plasmid was normalized to that of Renilla luciferase.

## RNA pull-down assay

Extraction of 100 μg total RNA from DMS-53 and SHP-77 cells was mixed with 100 μl streptavidin magnetic beads with 200 pmol biotin-labeled miR-145 mimic. The mixture was shaken for 30 min at 4-degree. After washing with cell lysis buffer, the associated RNA complexes in the beads were collected by Trizol reagent. CircMYC and MMP2 mRNA levels were quantified by RT-qPCR.

## Western blot

Protein concentration of total cell lysates was detected by BCA Protein assay kit (Beyotime Biotechnology; Shanghai, China). Ten percent SDS-PAGE was used for the electrophoretic separation of 20 µg proteins of each sample, and proteins were transferred to PVDF membranes by electroblotting. After blocking with 5% skimmed milk for 1 hour, the membrane was then incubated with primary antibodies at 4°C overnight. After washing with TBST buffer, HRP-labeled secondary antibodies were incubated at room temperature for 1 h. Then the membrane was washed four times with TBST, and the protein bands were visualized using an enhanced chemiluminescence kit (Santa Cruz, TX, USA) and photographed on a gel imager system (Bio-Rad, Hercules, CA, United States). The densitometry analysis was performed with the Image J software (Bethesda, MD, USA). Antibodies for Western blotting are as follows: PCNA (1:1000, ab29, Abcam, USA), Snail1 (1:1000, ab216347, Abcam, USA), MMP2 (1:1000, CA719E3C, Thermofisher Scientific, USA), and GAPDH (1:1000, 60,004, Proteintech, USA).

## CCK-8 experiments and clonogenic assays

DMS-53, SHP-77 cells in the logarithmic growth phase were seeded in 96 well plates at a density of 1 × 10^5^ cells per well. Ten microliters of CCK-8 solution was added at the indicated time point, and the cells were incubated in the incubator for 4 h. The absorbance at 450 nm was measured with a microplate reader. Experiments were repeated 3 times.

For colorogenic assay, cells were seeded into a 6-well plate (2000 cells/well), and the culture medium was changed every 3 days for 2 weeks. Cells were fixed with 4% paraformaldehyde at room temperature for 10 mins and stained with 0.5% crystal violet (Beyotime, Shanghai, China) for 20 mins. The number of colonies was counted under Leica AM6000 microscope (Leica, Wetzlar, Germany).

## Flow cytometry assay

The apoptosis analysis was detected by flow cytometry analysis. The detection of cell apoptosis was performed using the FITC Annexin V Apoptosis Detection Kit (BD Biosciences, PharMingen, San Jose, CA, USA) according to the manufacturer’s instructions. In brief, 1 μL Annexin V-FITC and 1 μL PI were added to the 1000 μL cell resuspension with 1 million cells for 30 mins in the dark. After washing twice with binding buffer, and cells were resuspended in 400 μL binding buffer and analyzed on BD FACS CantoTM II Flow Cytometer (BD Biosciences)

## Transwell migration and invasion assay

The Transwell (Corning, USA) was used to examine the migration and invasion ability in different transfection groups (si-NC, si-cirMYC) of DMS-53 and SHP-77 cells. For migration assays, 200 μL of cell suspension containing 1 × 10^5^ cells in a serum-free medium was added to the upper chamber, and 750 μL of RPMI 1640 medium containing 10% FBS was added to the lower chamber. After 24 h incubation, the upper chamber medium was discarded by aspiration, cells in the upper chamber were washed three times with PBS, fixed with 4% paraformaldehyde for 15 min and stained with crystal violet for 10 min. Migrating cells were counted in five randomly selected fields under a 100× inverted microscope. For invasion assay, the bottom membrane of the Transwell chamber was coated with 50 mg/L Matrigel diluted 1:4 in serum-free 1640 medium in advance.

## Binding site prediction analysis

Binding site prediction was performed using online bioinformatic tools: Circular RNA Interactome (https://circinteractome.nia.nih.gov/) and Starbase (http://starbase.sysu.edu.cn/) database.

## Statistical analysis

SPSS 22.0 statistical software was used for statistical testing. Measurement data were expressed as mean ± standard deviation. Normality checking has been performed using descriptive statistical functions of SPSS. A two-sample t test was used for comparison between two groups. Comparisons among multiple groups were analyzed using one-way analysis of variance (ANOVA) with Tukey’s post hoc test for pairwise comparison. Comparisons of data at multiple time points were examined using two-way ANOVA. For the overall survival analysis, SCLC patients were divided into high-expression and low-expression based on the median expression value of circMYC. Kaplan Meier Curve and log-rank test (p-value) were used to compare the cumulative survival rate. P < 0.05 was considered statistically significant, and all experiments were repeated three times.

## Results

In this study, the expression and functional roles of circMYC in SCLC tissues and cells were examined. We reported that circMYC was highly expressed in SCLC tissues and cell lines. Knockdown of circMYC inhibited cell proliferation, migration and invasion and induced apoptosis of SCLC. Mechanistically, circMYC upregulates the expression of MMP2 via targeting miR-145.

## CircMYC is highly expressed in SCLC tissues and cell lines

**Figure 1.**
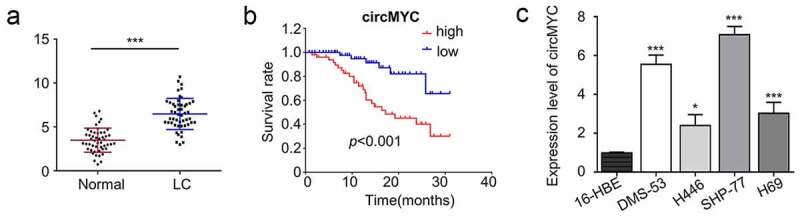
Relative expression level of circMYC in SCLC tissues and cell lines. (a) Expression levels of circMYC in 50 pairs of SCLC tissues and corresponding adjacent normal tissues. (b) KM-plotter plots survival curves of patients with circMYC high expression (n = 25) and low expression (n = 25) SCLC tissues. (c) Expression levels of circMYC in SCLC cell lines (DMS-53, H446, SHP-77, H69) and normal cell (16-HBE) cell lines. *P < 0.05. **P < 0.01. ***P < 0.001.

In this study, the expression level of circMYC was analyzed in 50 pairs of SCLC tissues and corresponding adjacent noncancerous tissues using qRT-PCR, and it showed that the expression of circMYC in SCLC tissues was remarkably higher than that in adjacent normal tissues (Figure 1a). The survival curves of circMYC high expression (n = 25) and low expression (n = 25) SCLC patients were plotted using KM-plotter, and patients with high circMYC expression had a poor prognosis (Figure 1b). In addition, the expression of circMYC was also analyzed in four SCLC cell lines (DMS-53, H446, SHP-77, and H69) and one normal human bronchial epithelial (16-HBE) cell line. The expression of circMYC was significantly increased in all four SCLC cell lines compared with 16-HBE, and the DMS-53 and SHP-77 cell lines showed the highest levels of circMYC expression (Figure 1c). These results suggested that circMYC is upregulated in SCLC tissue and cell lines.

## Knockdown of circMYC inhibits proliferation, migration, and invasion and induced apoptosis in SCLC cells

**Figure 2.**
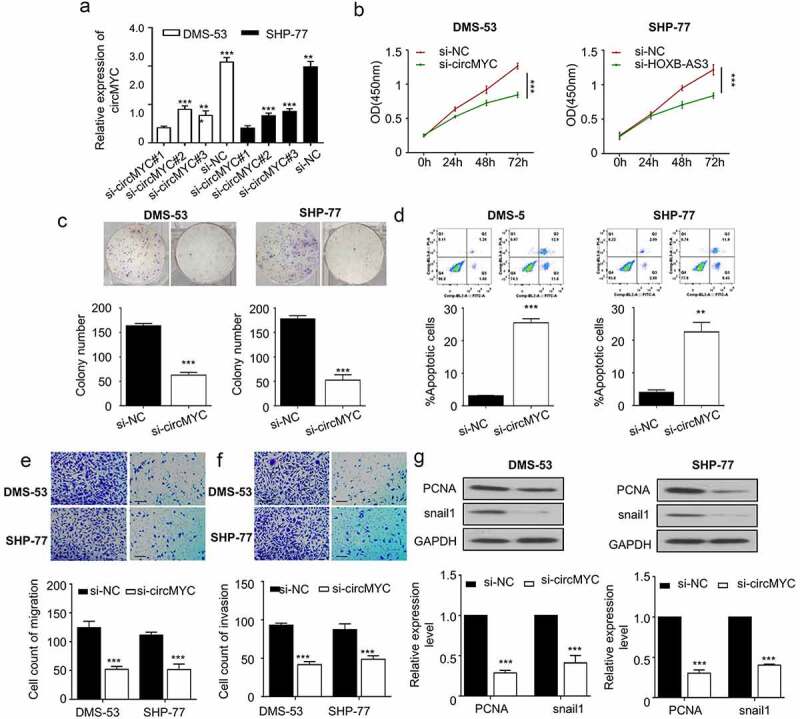
Knockdown of circMYC inhibits proliferation, migration invasion and induces apoptosis in SCLC cells. (a) Knockdown of circMYC in DMS-53 and SHP-77 cell lines by using SiRNA Si-circMYC. (b) CCK8 method was used to detect the absorption values at the wavelength of 450 nm in different transfection groups (si-NC, si-circMYC) of DMS-53 and SHP-77 cells for 0 h, 24 h, 48 h, 72 h. (c) clonogenic assay was performed to detect the clonogenic ability in different transfection groups (si-NC, si-circMYC) of DMS-53 and SHP-77 cells. (d) flow cytometry was used to detect the apoptosis level in different transfection groups (Si-NC, Si-circMYC) of DMS-53 and SHP-77 cells. (e) migration assay was performed to detect the migration ability in different transfection groups (si-NC, si-circMYC) of DMS-53 and SHP-77 cells. (f) invasion assay was performed to detect the invasion ability in different transfection groups (si-NC, si-circMYC) of DMS-53 and SHP-77 cells. (g) the protein levels of PCNA and Snail1 in different transfection groups (si-NC, si-circMYC) of DMS-53 and SHP-77 cells were detected by Western blot. *P < 0.05. **P < 0.01. ***P < 0.001.

Since circMYC was highly expressed in DMS-53 and SHP-77 cell lines, we subsequently used these two cell lines to further investigate the roles of circMYC during small cell lung carcinogenesis. We designed three siRNAs specifically targeting the splicing sequence of circMYC (si-circMYC#1, #2, and #3) and transfected them into DMS-53 and SHP-77 cell lines, and the knockdown efficiency was measured by qRT-PCR. Compared with the negative control si-NC, si-circMYC #1, #2, and #3 were able to efficiently knock down more than 50% of circMYC in DMS-53 and SHP-77 lines. Among these three siRNAs, si-circMYC #1 revealed the highest knockdown efficiency and was used in subsequent studies and named si-circMYC (Figure 2a). To explore the effect of circMYC on the proliferation ability of SCLC cell lines, the absorbance values of different transfection groups (si-NC, si-circMYC) of DMS-53 and SHP-77 cells at the wavelength of 450 nm for 0 h, 24 h, 48 h, 72 h were detected using CCK8 assay, and we found that knockdown of circMYC could significantly inhibit the proliferation of SCLC cells (Figure 2b). Meanwhile, a clonogenic assay was performed to detect the clonogenic ability in different transfection groups (si-NC, si-circMYC) of DMS-53 and SHP-77 cells. Knockdown of circMYC also effectively inhibited the clonogenic ability of the cells (Figure 2c). Flow cytometry was used to determine the level of apoptosis in different transfection groups (si-NC, si-circMYC) of DMS-53 and SHP-77 cells. Knockdown of circMYC significantly elevated the level of apoptosis (Figure 2d). A migration assay was performed to detect the migration ability in different transfection groups (si-NC, si-circMYC) of DMS-53 and SHP-77 cells. Knockdown of circMYC effectively inhibited the cell migration ability (Figure 2e). An invasion assay was performed to detect the invasion ability in different transfection groups (si-NC, si-circMYC) of DMS-53 and SHP-77 cells. Knockdown of circMYC inhibited the cell invasion ability (figure 2f). The knockdown of circMYC also significantly decreased the protein levels of proliferating cell nuclear antigen (PCNA) and Snail1 in DMS-53 and SHP-77 cells (Figure 2g). These findings revealed a close relationship between circMYC expression level and cell proliferation, apoptosis, migration, and invasion in SCLC cells, suggesting an oncogenic role of circMYC in SCLC cells.

## CircMYC targets miR-145

**Figure 3.**
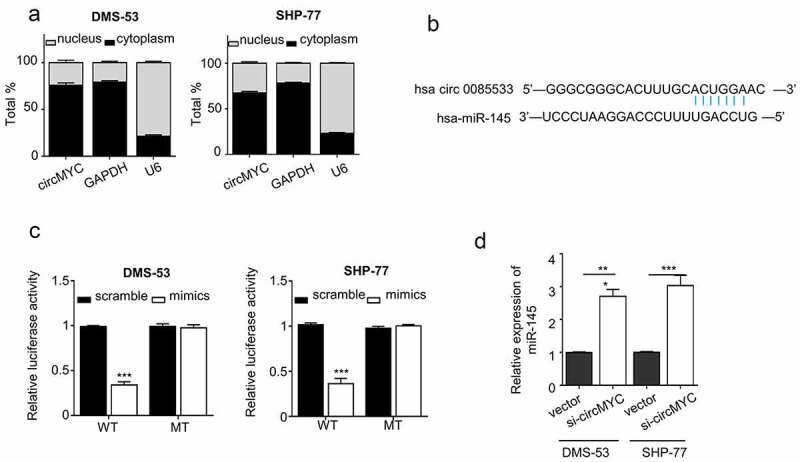
CircMYC targets miR-145. (a) Nuclear-cytoplasmic fractionation experiments of DMS-53 and SHP-77 cell, QRT-PCR was used to detect the expression levels of circMYC in the nucleus and cytoplasm, U6 and GAPDH were the internal reference of the nucleus and cytoplasm, respectively. (b) CircMYC was found to have binding sites with miR-145 by circinteractome prediction. (c) Luciferase reporter gene experiment validation. (d) QRT-PCR was performed to detect the miR-145 expression level change after knocking down circMYC. *P < 0.05. **P < 0.01. ***P < 0.001.

To investigate the mechanism of circMYC in SCLC tumorigenesis, it is necessary to investigate the location of circMYC in cells. We performed nucleocytoplasmic fractionation experiments in DMS-53 and SHP-77 cells to examine the cellular sub-localization of circMYC. A qRT-PCR assay was performed to examine the expression levels of circMYC in the nucleus and cytoplasm, and it was shown that circMYC was mainly localized and enriched in the cytoplasm (Figure 3a). It is acknowledged that circRNAs play their functional roles by interacting with miRNAs, we therefore used CircInteractome to predict the target miRNAs of circMYC and found that there was a potential binding site of miR145 in the sequence of circMYC (Figure 3b). To verify whether circMYC targets miR-145, we performed luciferase reporter assays in DMS-53 and SHP-77 cells. Overexpression of miR-145 was able to inhibit the luciferase activity of the reporter containing WT binding site when compared with the control group. However, the inhibitory effect was abolished after mutating the predicted miR-145 binding site in the MUT reporter (Figure 3c). qRT-PCR was performed to detect the expression level of miR-145 after knocking down circMYC in DMS-53 and SHP-77. Compared with Si-NC, knocking down of circMYC significantly elevated the expression of miR-145 (Figure 3d). These results demonstrated that circMYC could directly target on miR-145 and could inhibit the expression level of miR-145.

## MiR-145 targets MMP2 mRNA

**Figure 4.**
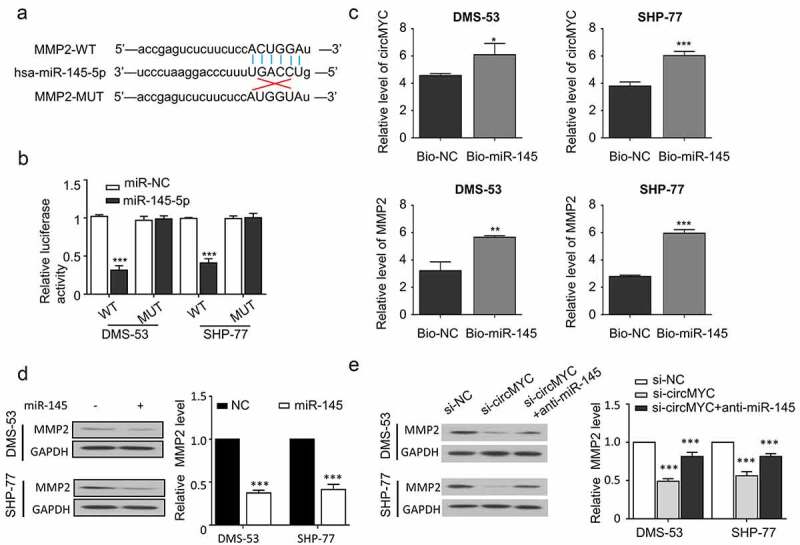
MiR-145 targeted MMP2 mRNA. (a) Starbase predicted binding sites between miR-145 and MMP2 mRNA. (b) Luciferase reporter assay verified the binding of miR-145 to MMP2 mRNA. (c) RNA pull down assay was performed to detect the binding of miR-145 to circmyc and MMP2 mRNA. (d) Protein expression levels of MMP2 after overexpression of miR-145 in DMS-53 and SHP-77 cells. (e) Western blot detected MMP2 protein levels in different transfection groups (si-NC, si-circMYC, si-circMYC+anti-miR-145). *P < 0.05. **P < 0.01. ***P < 0.001.

To further investigate the mechanisms of circMYC-miR-145 in SCLC, we predicted the downstream target genes of miR-145, and we found that there was a potential binding site between miR-145 and the 3ʹUTR of MMP2 mRNA (Figure 4a). Luciferase reporter assays in DMS-53 and SHP-77 cells were performed to verify the interaction between miR-145 and MMP2 mRNA. Compared with miR-NC, overexpression of miR-145 was able to inhibit the luciferase activity of WT reporter containing wild-type binding site, and its inhibitory effect was abolished when the predicted site was mutated in MUT reporter (Figure 4b). RNA pull-down assay was performed to validate the physical interaction between miR-145 and circMYC, as well as between miR-145 and MMP2 mRNA in DMS-53 and SHP-77 cells. Biotin-labeled miR-145 was able to enrich more circMYC and MMP2 mRNA as compared with Bio-NC (Figure 4c). The overexpression of miR-145 downregulated MMP2 expression at protein level (Figure 4d). The knockdown of circMYC also decreased the expression level of MMP2 protein, which was partially rescued when anti-miR-145 was co-transfected (Figure 4e). These results indicate that MiR-145 targets MMP2 mRNA to inhibit its expression.

## CircMYC regulates proliferation, migration invasion, and apoptosis of small cell lung cancer cells via MMP2

**Figure 5.**
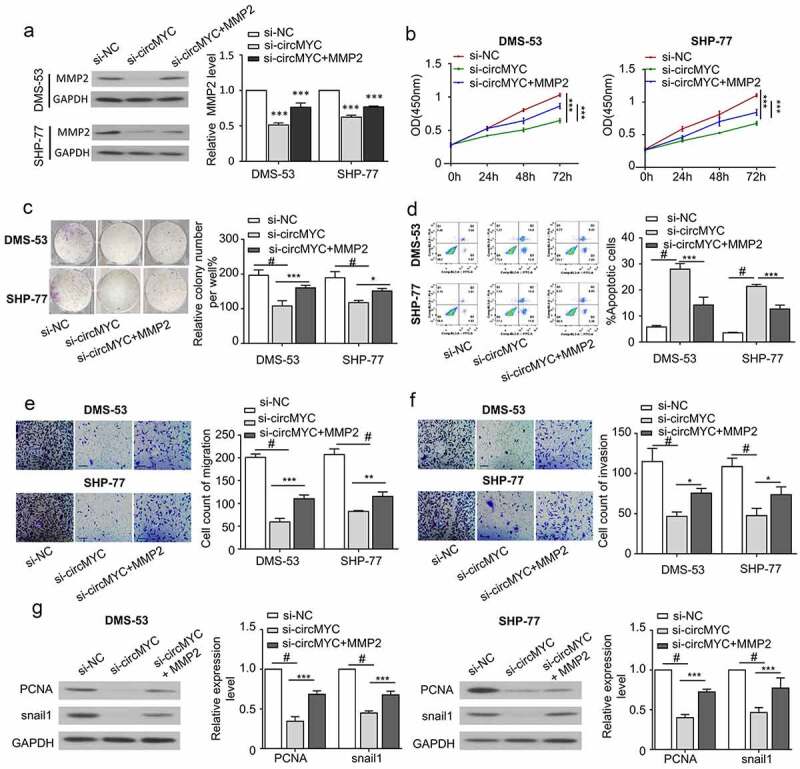
Circmyc regulates proliferation, migration invasion and apoptosis of SCLC cells through MMP2. (a) The protein expression levels of MMP2 in different groups (si-NC, si-circMYC, si-circMYC+MMP2) of dms-53 and shp-77 cells. (b) CCK-8 assay was performed in different groups (si-NC,si-circMYC, si-circMYC+MMP2) of dms-53 and shp-77 cells for 0 h, 24 h, 48 h, 72 h. (c) Clonogenic assay was performed to detect the clonogenic ability changes of different groups (si-NC, si-circMYC, si-circMYC+MMP2) of DMA-53 and SHP-77 cells. (d) Flow cytometry was used to detect the cell apoptosis. (e) Migration assay to detect the migration ability in different groups (si-NC, si-circMYC, si-circMYC +MMP2) of DMA-53 and SHP-77 cells. (f) Invasion assay to detect the invasion ability in different groups (si-NC, si-circMYC, si-circMYC +MMP2) of DMA-53 and SHP-77. (g) Western blot to detect PCNA and Snail1 protein levels in different groups (Si-NC, si-circMYC, si-circMYC+MMP2). *P < 0.05. **P < 0.01. ***P < 0.001.

To study the role of MMP2 in the mediation of circMYC function, we transfected SCLC cells with si-NC, si-circMYC, or Si-circMYC and MMP2 expression vector. The knockdown of circMYC decreased MMP2 level and the co-transfection of MMP2 vector was able to partially restore the protein level of MMP2 (Figure 5a). The CCK-8 proliferation assay revealed that, compared with si-NC, si-circMYC inhibited cell proliferation and MMP2 overexpression could partially rescue the proliferation in DMS-53 and SHP-77 cells (Figure 5b). The knockdown of circMYC also inhibited the clonogenic ability of SCLC cells, which was rescued by MMP2 overexpression (Figure 5c). In addition, the apoptosis induced by the knockdown of circMYC was suppressed by the co-transfection of the MMP2 plasmid (Figure 5d). MMP2 overexpression also partially rescued the migration and invasion ability upon circMYC silencing (Figure 5e and 5f). Western blot was performed to detect the protein levels of PCNA and Snail1 in different groups. The knockdown of circMYC suppressed the protein levels of PCNA and Snail1, and MMP2 overexpression partially elevated the protein levels of PCNA and Snail1 (Figure 5g). These data suggest that MMP2 mediates the functional roles of circMYC in SCLC cells.

## Discussion

To the best of our knowledge, this is the first study exploring the roles of circMYC in SCLC. In this work, we found that circMYC is highly expressed in SCLC tissues and cell lines, and our study in DMS-53 and SHP-77 cell lines support that circMYC is an oncogenic circRNA promoting the malignant phenotype of SCLC. In addition, we demonstrated that circMYC is mainly localized in the cytoplasm of SCLC cells and targets miR-145 to upregulate the expression of MMP2. The high level of MMP2 expression can support the proliferation, migration and invasion and inhibit apoptosis of SCLC cells. Overall, our study identified a novel axis of circMYC-miR-145-MMP2, which is potentially involved in the malignant progression of SCLC cells.

CircRNAs are relatively stable and have a longer half-life than the linear RNAs [23]. CircRNAs show a good conservation among species [24], in addition, the expression of circRNAs is tissue-specific and developmental stage-specific [25–27]. Recent studies also suggest that the dysregulation of circRNAs could serve as potential biomarkers for cancer management, such as in lung cancer [28–30]. A number of studies have demonstrated that multiple circRNAs can be used as independent prognostic indicators in lung cancer patients and are closely associated with the survival in lung cancer patients [29,31,32]. In this study, we found that circMYC is highly expressed in SCLC tissues and cell lines, which is associated with a poor prognosis in SCLC patients. These findings highlight the oncogenic role of circMYC in SCLC cells, which are consistent with previously reported tumor-promoting function of circMYC in myeloma, and nasopharyngeal carcinoma [20–22].

Apart from the diagnostic potential, circRNAs can also act as regulatory molecules to promote or inhibit the development of lung cancer, and targeting circRNAs may have important implications for the treatment of lung cancer [33–36]. CircRNAs can function as a molecular sponge for miRNAs and consequently repress the function of miRNAs [37,38]. Interestingly, our study revealed that circMYC also functions as a miRNA molecular sponge in SCLC cells, and circMYC acts as a ceRNA that competitively binds to miR-145 and inhibits miR-145 expression. We showed that circMYC is highly expressed in SCLC, whereas miR-145 was previously reported to be lowly expressed in multiple tumors, including SCLC [39–44]. CircMYC inhibits miR-145 expression and releases its inhibition on the expression of downstream target MMP2. It has been documented that MMP2 is highly expressed in lung cancer and closely associated with a poor prognosis of patients [45–47]. However, it remains unknown whether the interaction of circMYC with other miRNAs also regulates the malignancy of SCLC cells.

A remaining question is what is the mechanism underlying the upregulation of circMYC in SCLC tumor. It is of great importance to investigate the upstream signals or mechanisms modulating circMYC expression, which could provide insights into the manipulative strategies of circMYC expression. CircMYC is derived from the *myc* gene, which is a canonical oncogene [48,49]. CircMYC is co-transcriptionally produced with myc mRNA and is subject to the same regulation at the transcriptional level. Whether circMYC and myc mRNA potentially interact with each other to coordinate biological functions remains to be studied.

## Conclusions

Our study showed that circMYC is highly expressed in SCLC tumor, which is required to support the malignant phenotype of SCLC cells by promoting the expression of MMP2. This regulation is mediated by the sponging effect of circMYC on miR-145. These findings indicate that targeting circMYC/miR-145/MMP-2 axis could serve as a potential intervention for SCLC treatment.

## Data Availability

The data is available from the corresponding author on reasonable request.
